# Laparoscopic partial nephrectomy for the horseshoe kidney with indocyanine green fluorescence guidance under the modified supine position

**DOI:** 10.1002/iju5.12450

**Published:** 2022-04-26

**Authors:** Yu Imai, Fumihiko Urabe, Wataru Fukuokaya, Akihiro Matsukawa, Kosuke Iwatani, Koichi Aikawa, Koki Obayashi, Takafumi Yanagisawa, Shunsuke Tsuzuki, Hiroshi Nakajo, Takahiro Kimura, Shin Egawa, Jun Miki

**Affiliations:** ^1^ Department of Urology The Jikei University School of Medicine, Kashiwa Hospital Kashiwa Chiba; ^2^ Department of Urology The Jikei University School of Medicine Tokyo Japan

**Keywords:** Horseshoe kidney, indocyanine green, laparoscopic partial nephrectomy, modified supine position, renal cell carcinoma

## Abstract

**Introduction:**

Owing to the complexity of their blood supply, renal tumors in horseshoe kidneys are sometimes technically challenging to resect through laparoscopic procedures.

**Case presentation:**

A 75‐year‐old man presented with a 3‐cm lower‐pole mass in the right moiety of the horseshoe kidney. Indocyanine green administration allowed for the identification of the tumor's feeding artery, which was selectively clamped to perform laparoscopic partial nephrectomy. During the procedure, the patient was positioned in the modified supine position (30° semi‐lateral position), which enabled us to approach the branch of the left renal artery. Postoperative pathologic examination of the resected mass confirmed the diagnosis of pT1a clear cell renal cell carcinoma with negative surgical margins.

**Conclusion:**

Our novel laparoscopic approach with indocyanine green fluorescence in the modified supine position facilitates the identification of and access to the tumor's feeding artery. This technique is advantageous for laparoscopic partial nephrectomy in patients with horseshoe kidney.

**Abbreviations & Acronyms**
CECT: contrast‐enhanced computed tomography
CT: computed tomography
HSK: horseshoe kidney
ICG: indocyanine green
NIRF: near‐infrared fluorescence
LPN: laparoscopic partial nephrectomy
RAPN: robot‐assisted partial nephrectomy
RCC: renal cell carcinoma
WIT: warm ischemia time

**Keynote message**

We performed laparoscopic partial nephrectomy with guidance from indocyanine green fluorescence in a modified supine position for a renal mass in a horseshoe kidney.

## Introduction

HSK is the most common congenital fusion anomaly of kidney, and its prevalence within the world population ranges approximately from 0.15% to 0.25%.^1^, ^2^ HSK is characterized by unique anatomical features, such as a complex blood supply. Here, we present the case of a patient with a renal mass located in a HSK, which was successfully treated by LPN with guidance from ICG fluorescence in the modified supine position.

## Case presentation

**Fig. 1:**
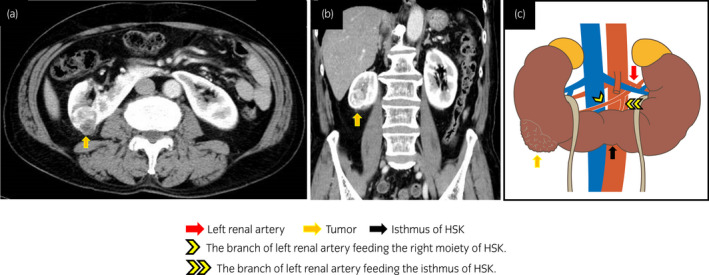
(a, b) Imaging findings of a right renal tumor in horseshoe kidney (HSK). (c) Schematic representation of the tumor's feeding vessels. [Colour figure can be viewed at wileyonlinelibrary.com]

A 75‐year‐old man with HSK was referred to our department due to a 3‐cm lower‐pole tumor, which was incidentally discovered by CECT in the posterior part of the right side of the kidney (Fig. 1a). The patient was diagnosed with cT1aN0M0 RCC (RENAL Nephrometry Score: 1 + 2 + 2 + 1 = 6p, Fig. 1a,b). CECT also revealed that the right moiety of the HSK was supplied by the right renal artery and the branch of the left renal artery, which also perfused the isthmus (Fig. 1c).

**Fig. 2:**
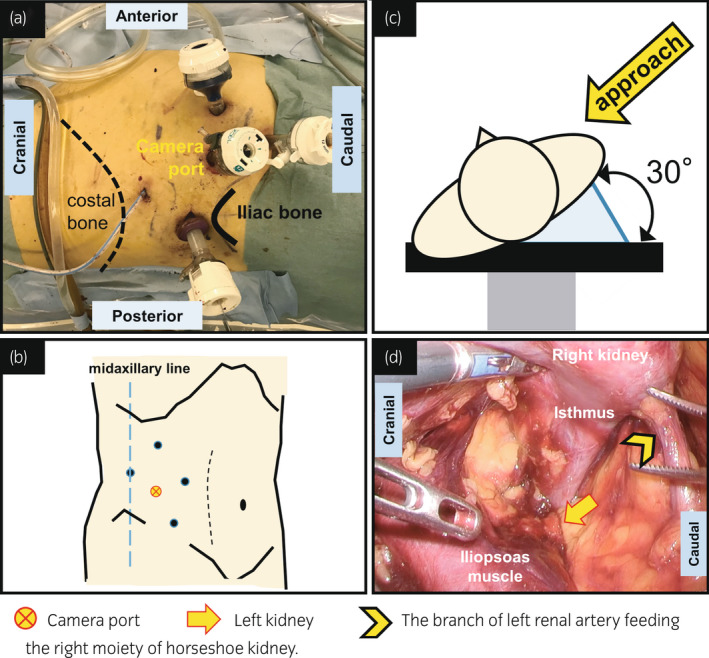
(a, b) Port placement for laparoscopic partial nephrectomy. The camera port placement was more caudal and ventral than conventional retroperitoneal approach. (c) The modified supine position. d) Laparoscopic view of the posterior surface of the isthmus. [Colour figure can be viewed at wileyonlinelibrary.com]

We performed retroperitoneal LPN in the modified supine position (30° semi‐lateral position) with five ports (5–12 mm) (Fig. 2a–c). During the procedure, the camera port was placed at a more caudoventral position than the usual placement preferred during the conventional retroperitoneal procedure (Fig. 2a,b). This position for the port renders the approach to the distal isthmus easy when the patient is in the modified supine position. Additionally, in our patient, CECT showed the arteries supplying the right kidney and the isthmus would originate from the left renal artery (Fig. 1c), thus it was necessary to identify the vessel feeding the tumor before tumor resection could be performed. To approach the branch of the left renal artery, both the anterior and posterior surfaces of the kidneys have to be exposed, especially the front and back of the isthmus. In light of this, the modified supine position and camera port placement helped expose the retroperitoneal cavity and distal isthmus (Fig. 2d).

**Fig. 3:**
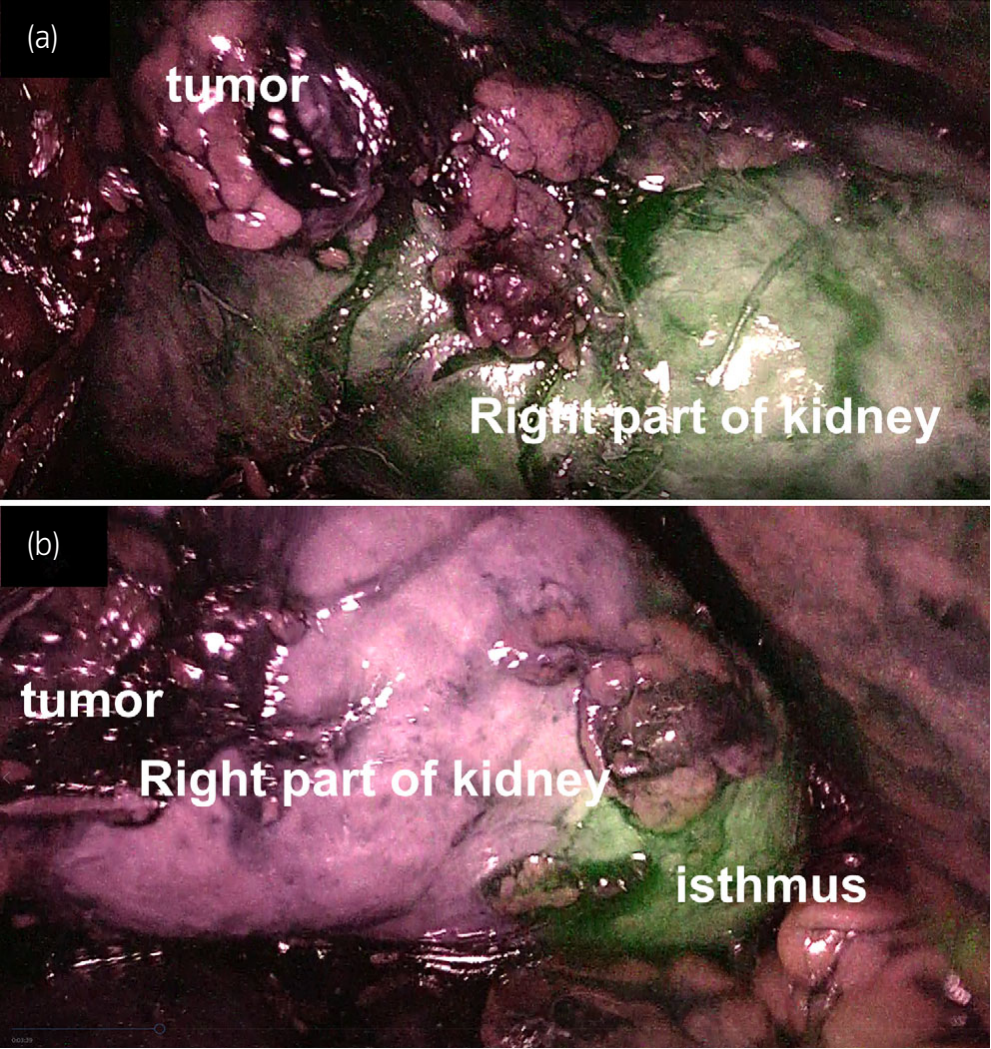
**(**a) Indocyanine green (ICG)‐based near‐infrared fluorescence (NIRF) images showing strong ICG fluorescence in the right part of the kidney, except in the tumor. (b) The absence of fluorescence in the right part of kidney after clamping of the right renal artery. [Colour figure can be viewed at wileyonlinelibrary.com]

A NIRF system with was employed to identify the tumor's feeding artery. One minute after intravenous administration of 1 mL of 2.5 mg/mL ICG, strong fluorescence was observed in the right part of the kidney except in the tumor (Fig. 3a). When the right renal artery was selectively clamped, fluorescence disappeared from the right kidney (Fig. 3b). These findings indicated that only the right renal artery supplied blood to the tumor and the surrounding renal parenchyma, which allowed us to clamp the right renal artery selectively.

The tumor was resected following a standard protocol, and the resected site was closed by a running suture using 2–0 V‐Loc™ (Medtronic, Minneapolis, MN, USA). The operation time was 225 min, and the WIT was 18 min. The patient was discharged without any intraoperative or postoperative complications. The pathological diagnosis of the resected specimen was pT1a clear cell RCC (World Health Organization/International Society of Urological Pathology [WHO/ISUP] grade 1) with negative surgical margins. No local recurrence or metastasis was observed with follow‐up CT, and there was no change in renal function at 3 months.

## Discussion

One of the unique anatomical features of HSK tumors is aberrant blood vessels in the isthmus that can bifurcate from the aorta, the common, internal or external iliac artery, or from the inferior mesenteric or sacral arteries.^3^ The likelihood of the presence of aberrant blood vessels is 60%.

One novel aspect of our study is the employment of the modified supine position, which was deemed as the optimal surgical placement of the patient because it allowed to visualize the anterior aspects of the isthmus for the exact identification of the vessel supplying the tumor and its surroundings. In fact, reaching the anterior aspects of the isthmus is likely to be challenging with the conventional retroperitoneal approach in the lateral position.^4^ On the contrary, the modified supine position provides for better visualization of the anterior surface of the kidney and isthmus.^5^

Another innovative aspect of this report is the use of ICG‐guided NIRF. This technique has been applied in various clinical settings. Especially, the NIRF imaging technology, which offer real‐time fluorescing information, has been applied to a variety of surgical procedures, such as for evaluation of vessel blood flow, identification of sentinel lymph nodes, and detection of cancerous masses.^6^, ^7^, ^8^, ^9^

Injection of ICG, which binds to serum proteins, enables surgeons to visualize the vascular network with fluorescence.^10^, ^11^ While normal parenchyma is generally isofluorescent, most tumors are hypofluorescent due to the absence of bilitranslocase, an ICG carrier protein that is instead present in normal proximal tubule cells.^12^ Because of this, ICG‐based NIRF can distinguish malignant from nonmalignant tissues.

To date, the usefulness of ICG fluorescence in laparoscopic and robotic urologic procedures has been reported.^13^, ^14^ However, no report evaluated its utility in HSK. In renal tumors arising from HSK, it is important to decide which artery should be selectively clamped, as HSK can display a unique vascular network. The isthmus and adjacent parenchymal of masses may be supplied by a single renal artery or multiple arteries. In addition, accessary arteries can originate from aorta.^15^ In this case, fluorescence was observed in the right renal parenchyma, except in the tumor, while no fluorescence was visible in this area after selective clamping of the right renal artery, which was very informative to determine the tumor's margin and select the feeding artery to be clamped.

**Fig. 4:**
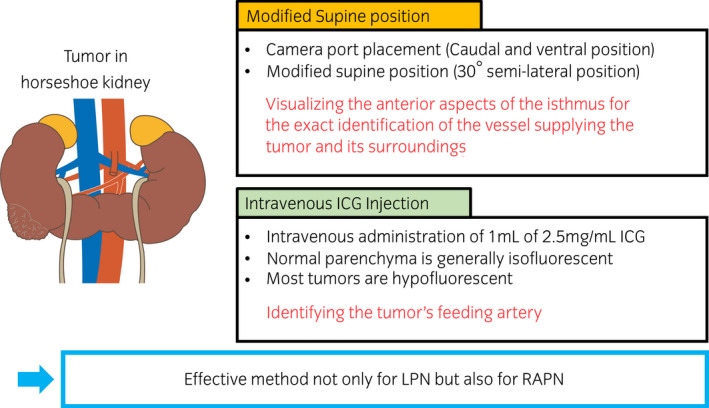
Summary of our novel laparoscopic approach with indocyanine green fluorescence in the modified supine position. [Colour figure can be viewed at wileyonlinelibrary.com]

ICG guidance LPN for HSK was a very effective technique for assessing blood flow and identifying the area of blockage. In addition, the semi‐lateral positioning and modified port placement provided an excellent operative field. It is considered to be an effective method not only for LPN but also for RAPN (Fig. 4).

## Conflict of interest

The authors declare no conflict of interest.

## Approval of the research protocol by an Institutional Reviewer Board

Not applicable.

## Informed consent

Consent to participate and for publication were acquired from the patient.

## Registry and the registration no. of the study/trial

Not applicable.

## Author Contributions

Yu Imai: Conceptualization; data curation; writing – original draft. Fumihiko Urabe: Conceptualization; data curation; writing – original draft; writing – review and editing. Wataru Fukuokaya: Writing – review and editing. Akihiro Matsukawa: Conceptualization; writing – review and editing. Kosuke Iwatani: Conceptualization; visualization; writing – review and editing. Koichi Aikawa: Conceptualization; visualization. Koki Obayashi: Writing – review and editing. Takafumi Yanagisawa: Supervision; writing – review and editing. Shunsuke Tsuzuki: Supervision; writing – review and editing. Hiroshi Nakajo: Supervision; writing – review and editing. Takahiro Kimura: Conceptualization; supervision; writing – review and editing. Shin EGAWA: Supervision; writing – review and editing. Jun Miki: Conceptualization; data curation; investigation; methodology; supervision; visualization; writing – original draft; writing – review and editing.
